# A comparative study of pseudo-dipole arrays and standard arrays based on the high-density DC resistivity method

**DOI:** 10.1371/journal.pone.0360161

**Published:** 2026-10-14

**Authors:** Xiaoyi Jiang, Shuhai Jiang, Hui Chen, Jianjun Gong, Suiming Liu

**Affiliations:** 1 Zhejiang Institute of Hydraulics & Estuary (Zhejiang Institute of Marine Planning & Design), Hangzhou, Zhejiang, China; 2 Zhejiang Guangchuan Engineering Consulting Co., Ltd., Hangzhou, Zhejiang, China; 3 East China University of Technology, Nanchang, Jiangxi, China; 4 Xinjiang Institute of Technology, Urumqi, Xinjiang, China; Zhejiang A&F University, CHINA

## Abstract

High-density direct current resistivity surveys play a crucial role in characterizing aquifer structures and groundwater contamination. However, the necessity of using multiple electrode arrays to achieve comprehensive subsurface imaging significantly prolongs field data acquisition, posing challenges to efficient hydrological investigations. This study validates an efficient approach that employs a single pseudo-dipole array to derive comprehensive datasets equivalent to those obtained from multiple standard arrays (e.g., Wenner α, β, γ). The pseudo-dipole configuration, which utilizes fixed reference electrodes positioned at the line ends, demonstrates vertical resolution comparable to that of conventional dipole arrays, while offering superior lateral resolution in our analysis. Numerical modeling and analytical solutions for homogeneous half-space, vertical contact, and multi-anomaly models confirmed that the pseudo-dipole array exhibits enhanced sensitivity to subsurface anomalies. Numerical experiments demonstrate that high-resolution apparent resistivity pseudosections and reliable inversion models, derived from transformed pseudo-dipole data, can effectively identify hydrological units. This method provides a time-efficient solution for detailed aquifer mapping and contamination plume monitoring, thereby enhancing the utility of electrical resistivity tomography in water resource management.

## 1. Introduction

The high-density direct current resistivity method has become a cornerstone technique in shallow geophysical exploration due to its notable advantages, including straightforward field procedures, high operational efficiency, dense spatial data coverage, and the ability to yield comprehensive geoelectrical information. Its broad applicability spans multiple domains, such as hydrogeological surveys, characterization of groundwater occurrence conditions, delineation of aquifer structures, detection of dam leakage, and engineering geological assessments related to water resource management [1–5]. Since the introduction of array-based electrical prospecting in the 1970s, the method has evolved significantly through sustained technological innovation, leading to the establishment of standardized electrode array configurations—such as dipole-dipole, pole-pole, Wenner, and Schlumberger arrays. Each configuration produces apparent resistivity profiles with distinct characteristics in terms of depth of investigation and sensitivity to subsurface anomalies. By integrating data from multiple electrode arrays, exploration coverage is improved, blind zones are minimized, and the resolution and accuracy of inverse models for critical hydrogeological features—particularly aquifer architectures—are substantially enhanced [6–11].

In practical hydrogeological exploration, data acquisition in high-density direct current resistivity methods typically relies on the empirical selection of single or combined electrode arrays that exhibit enhanced sensitivity to water-bearing structures [12]. The theoretical foundation for array transformation was established by Xu and Noel [13], who demonstrated that observation data from any electrode configuration can be synthesized via linear combination using a complete dipole-dipole dataset. Building on this, Lehmann [14] developed methods for transforming such datasets into Schlumberger array data. Blome et al. [15] further demonstrated the theoretical completeness of dipole-dipole datasets, showing that they contain all measurable geoelectrical information and providing the mathematical justification for subsequent transformation approaches. More recently, Rucker [16], Rucker and Glaser [17] conducted systematic studies on converting dipole-dipole data into other high-resolution datasets, focusing on practical applications of array transformation.

To avoid confusion, it is crucial to clarify the distinction between the pseudo-dipole array investigated in this study and the pseudo-pole-pole array recently proposed by You and Butler [18]. While both configurations eliminate the need for remote “infinity” electrodes, they differ fundamentally in electrode arrangement. The pseudo-pole-pole array employs one fixed current electrode and one fixed potential electrode at opposite ends of the survey line, with the remaining electrodes serving as mobile dipoles. In contrast, our pseudo-dipole array fixes both a reference current electrode (C) and a reference potential electrode (P) at the terminal positions, with electrodes B (current) and M (potential) as the mobile pair. This configuration enables direct acquisition of a complete dataset that can be transformed into any standard array without the additional processing steps required for pseudo-pole-pole data. Furthermore, while previous work by Rucker and Glaser [17] focused on converting existing dipole-dipole data to other array types, our study addresses the complementary challenge of designing an acquisition configuration specifically optimized for subsequent transformation. The pseudo-dipole array presented here builds upon the theoretical foundations established by Blome et al. [15] while addressing the practical limitation of requiring remote electrodes—a point recently raised by You and Butler [18] for the pseudo-pole-pole configuration.

This study presents a systematic investigation of the pseudo-dipole electrode configuration and its potential applications in hydrological exploration, utilizing a geophysical forward and inverse modeling program developed by our research team [19]. By analyzing the principles of array transformation and the operational modes of the pseudo-dipole array, we compare its forward response characteristics and inversion outcomes with those of standard electrode arrays, assess its performance in resolving aquifer structures in both lateral and vertical dimensions, and conduct quality control analyses of the transformation process. Building on these foundations, this study makes three specific contributions: (1) we systematically evaluate the forward response characteristics and inversion performance of the pseudo-dipole array relative to conventional dipole and Wenner arrays, providing quantitative comparisons of lateral and vertical resolution; (2) we develop and validate a transformation method from pseudo-dipole measurements to standard Wenner (α, β, γ) array datasets, including a comprehensive error analysis under varying noise conditions; and (3) we establish quality control thresholds for data filtering during transformation, demonstrating that data with relative differences exceeding 0.2 should be excluded to maintain inversion reliability. The results show that the pseudo-dipole configuration achieves superior lateral resolution relative to conventional dipole arrays while preserving comparable vertical resolution. Furthermore, Wenner array data generated through transformation from pseudo-dipole measurements exhibit close agreement with directly acquired Wenner (α, β, γ) data in terms of apparent resistivity pseudosections and inverted models. These findings underscore the reliability and applicability of the pseudo-dipole approach in hydrogeophysical surveys, particularly for high-resolution aquifer characterization.

## 2. Principles of direct current resistivity exploration

Fig 1 presents a schematic illustration of resistivity measurement in a uniform isotropic half-space medium using the four-electrode method. In this configuration, electrodes A and B serve as the current injection terminals (positive and negative, respectively), while electrodes M and N function as the potential measurement electrodes (positive and negative, respectively). The apparent resistivity of the subsurface can be determined by injecting a known current into the ground through the AB pair and measuring the resulting potential difference between the MN pair.

**Fig 1:**
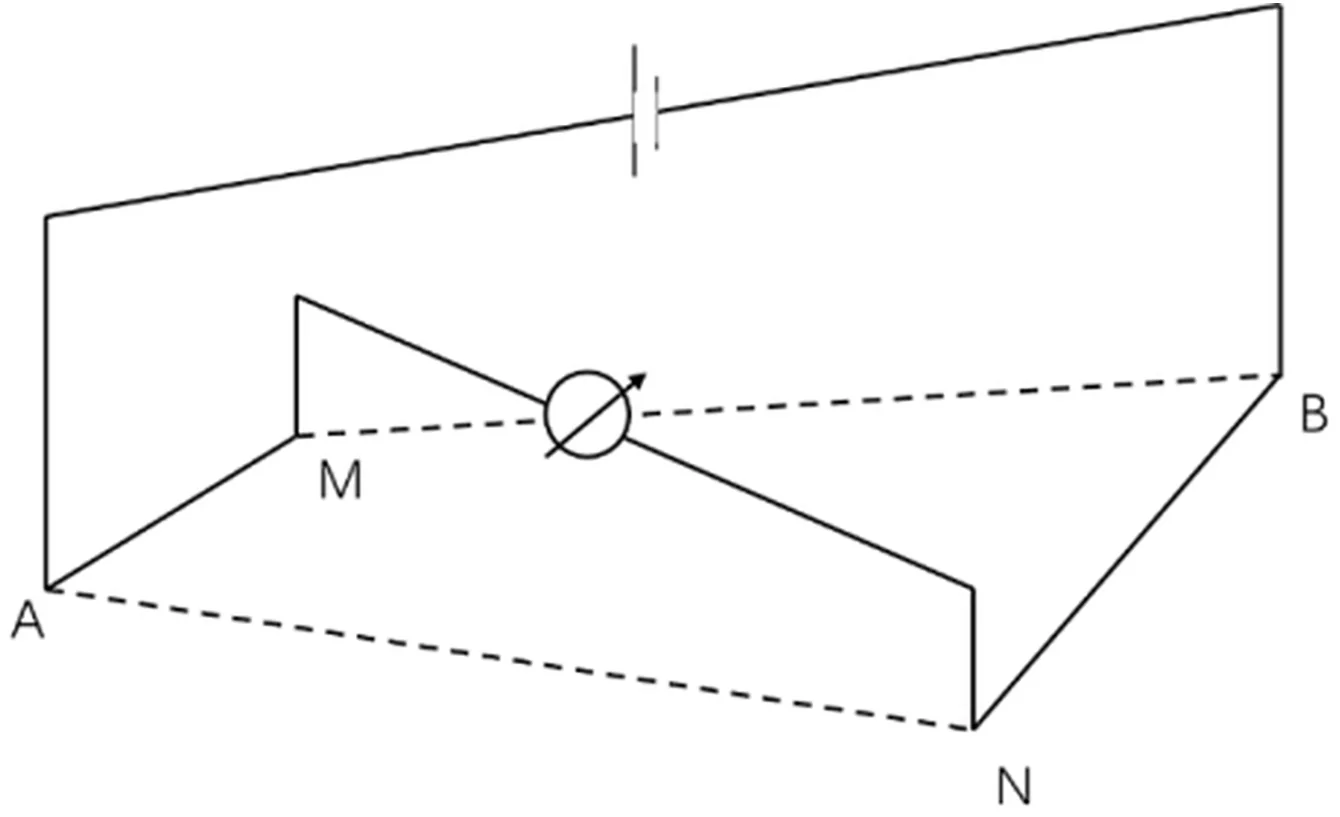
Schematic diagram of any four-electrode device.

Assuming the potentials at current electrodes A and B, as well as in the vicinity of potential electrodes M and N, are denoted by $U_{A}^{M}$, $U_{A}^{N}$, $U_{B}^{M}$ and $U_{B}^{N}$, respectively, and given an input current I, the following relationship holds:


$$\begin{array}{c} U_{A}^{M}=\frac{I\rho_{A,M}}{2\pi }\cdot \frac{\text{1}}{r_{AM}},U_{A}^{N}=\frac{I\rho_{A,N}}{2\pi }\cdot \frac{\text{1}}{r_{AN}},U_{B}^{M}=\frac{I\rho_{B,M}}{2\pi }\cdot \frac{\text{1}}{r_{BM}},U_{B}^{N}=\frac{I\rho_{B,N}}{2\pi }\cdot \frac{\text{1}}{r_{BN}} \end{array}$$ (1)

In the equation, $r_{AM}$, $r_{AN}$, $r_{BM}$, and $r_{BN}$ represent the electrode spacings corresponding to different potential measurement points, while $\rho_{A,M}$, $\rho_{A,N}$, $\rho_{B,M}$, and $\rho_{B,N}$ denote the resistivity values of the homogeneous half-space. By assuming the Earth to be a homogeneous half-space, the corresponding apparent resistivity can be defined.

Based on the principle of superposition of electric fields, the potential difference between points M and N can be determined as:


$$\begin{array}{c} U_{A,B}^{M,N}\text{=}U_{A,B}^{M}-U_{A,B}^{N}\text{=}\left(U_{A}^{M}-U_{B}^{M}\right)-\left(U_{A}^{N}-U_{B}^{N}\right) \end{array}$$ (2)

In the equation, the subscripts A and B of the symbol $U_{A,B}^{M,N}$ denote the current electrodes, while the superscripts M and N represent the potential electrodes. The notation A, M preceding the comma indicates the positive terminal of the current or potential electrode, whereas B, N following the comma denotes the negative terminal. Consequently, the apparent resistivity $\rho_{s}$ of the quadrupole configuration is expressed as follows:


$$\begin{array}{c} \rho_{s}=K\frac{\Delta U}{I} \end{array}$$ (3)

In the formula, the device coefficient. $K=2\pi /(\frac{\text{1}}{AM}-\frac{\text{1}}{AN}-\frac{\text{1}}{BM}+\frac{\text{1}}{BN})$

As indicated in Equation 2, given a complete dataset obtained from a dipole array, the potential of any other four-electrode configuration can be calculated based on the potentials measured from four dipole arrays. According to the definition of mutual resistance—which refers to the voltage observed at one electrode resulting from current injection into another electrode, with an equal voltage appearing on the connected electrode [20–24]—the following properties hold: (1) interchanging the positions of the current and potential electrodes does not affect the measured potential difference; (2) reversing the polarity of either the current or potential electrodes changes only the sign of the measurement, while the ratio R of potential to current across the electrode pair remains invariant.


$$\begin{array}{c} R_{C}^{P}=R_{P}^{C} \end{array}$$ (4)

In the equation, $R_{C}^{P}$ denotes the transfer resistance between the reference electrodes C and P [15,18]. For the transfer resistance $R_{1,4}^{2,3}$ in a four-electrode measurement, the following holds:


$$\begin{array}{c} R_{1,4}^{2,3}=-R_{1,4}^{3,2}=-R_{4,1}^{2,3}=R_{4,1}^{3,2} \end{array}$$ (5)

Based on the aforementioned properties, the following rule can be applied to combine two four-electrode arrays that share the same current electrodes but differ in their potential electrodes:


$$\begin{array}{c} R_{1,4}^{2,3}=R_{1,4}^{2,P}+R_{1,4}^{P,3} \end{array}$$ (6)

In the same way, two four-electrode arrays with the same potential but different current electrodes are:


$$\begin{array}{c} R_{1,4}^{2,3}=R_{4,C}^{2,3}+R_{C,1}^{2,3} \end{array}$$ (7)

In this paper, the reference current electrode of the pseudo-dipole array is positioned at the positive electrode, and the reference potential electrode is located at the negative electrode. Consequently, the transfer resistance formula for the four-electrode measurement, denoted as $R_{1,4}^{2,3}$ (with the Wenner α array used as an illustrative example), can be expressed as


$$\begin{array}{c} R_{1,4}^{2,3}=R_{1,4}^{2,P}+R_{1,4}^{P,3}=R_{C,1}^{3,P}-R_{C,1}^{2,P}-R_{C,4}^{3,P}+R_{C,4}^{2,P} \end{array}$$ (8)

In the same way,


$$\begin{array}{c} R_{1,4}^{2,3}=R_{C,1}^{2,3}+R_{C,4}^{2,3}=R_{C,1}^{3,P}-R_{C,1}^{2,P}-R_{C,4}^{3,P}+R_{C,4}^{2,P} \end{array}$$ (9)

The physical meaning of equations (8) and (9) can be understood as follows: For any four-electrode configuration (A, B, M, N), the measured potential difference ΔV_MN can be expressed as a linear combination of the potentials measured at electrodes M and N due to current injection at A and B. In practice, this means that once the complete dipole-dipole dataset is acquired—containing all possible potential measurements from current injections at all electrode pairs—any standard array configuration (Wenner, Schlumberger, etc.) can be reconstructed through weighted summation of the appropriate dipole measurements. The operational procedure involves: (1) identifying the four electrodes corresponding to the target configuration; (2) extracting the relevant dipole measurements from the complete dataset; (3) applying the linear combination coefficients specified by equations (8) and (9); and (4) computing the apparent resistivity using the geometric factor of the target array.

The process of transforming a formula into a standard (9)Wenner α array is shown in Fig 2:

**Fig 2:**
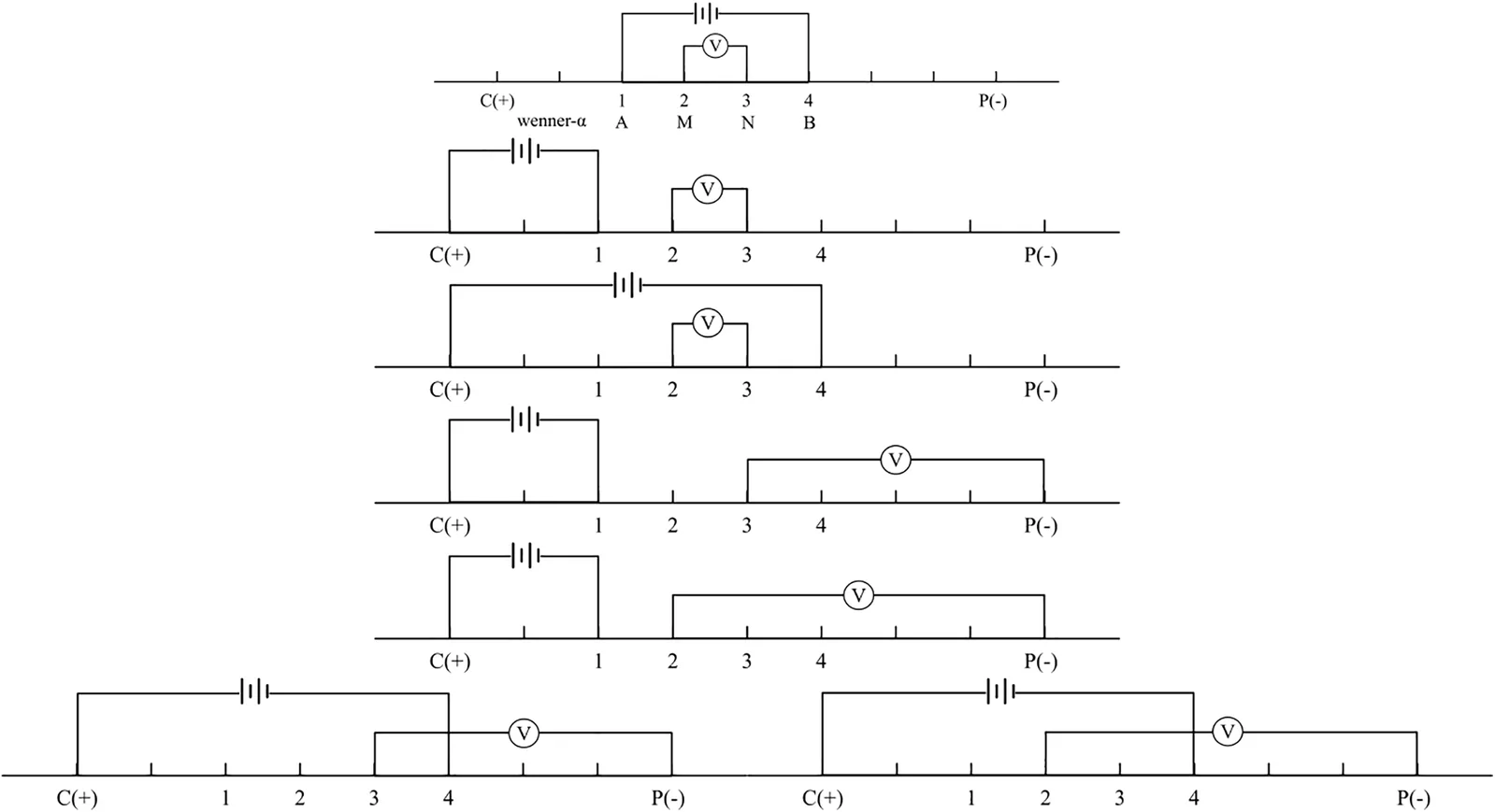
Schematic diagram of pseudo-dipole array conversion principle.

## 3. Operation of the pseudo-dipole array

The electrode configuration and operational mode of the pseudo-dipole array (α and β arrays) are described as follows. In the initial setup, the positive current electrode C (reference current electrode) is fixed at the starting end of the survey line, while the negative potential electrode P (reference potential electrode) remains fixed at the far end of the survey line. The negative current electrode B is positioned one electrode spacing away from C, and the positive potential electrode M is located two electrode spacings from C. During data acquisition, the separation between electrodes B and M is progressively increased by one electrode spacing at each measurement step, generating a slantwise sounding profile. Subsequently, both B and M are advanced simultaneously by one electrode spacing along the survey line to initiate a new sounding sequence. This scanning procedure is systematically repeated, ultimately forming an inverted trapezoidal measurement grid. The recording point for each measurement is defined as the midpoint of segment BM, with the apparent depth approximated as BM/2. In contrast to the pseudo-dipole α array, the β array involves an interchange of the positions of current electrode B and potential electrode M; however, the overall electrode movement pattern remains identical to that of the α array.

To provide a clearer understanding of how the pseudo-dipole array differs from other configurations, we offer the following comparative description. In contrast to the conventional dipole array, where both current and potential dipoles move independently along the survey line, the pseudo-dipole array maintains fixed reference electrodes at both ends. This fundamental difference in measurement geometry results in distinct data acquisition patterns and resolution characteristics. Compared to the pseudo-pole-pole array recently proposed by You and Butler [18], which fixes one current electrode and one potential electrode at opposite ends of the survey line, our pseudo-dipole array fixes both a reference current electrode (C) and a reference potential electrode (P) at the terminal positions. This configuration enables direct acquisition of a complete dataset that can be transformed into any standard array without the additional processing steps required for pseudo-pole-pole data. Table 1 summarizes these differences, providing a concise side-by-side comparison of the key features of each array type.

**Table 1:** Comparison of electrode array configurations.

Array Type	Fixed Electrodes	Moving Electrodes	Measurement Pattern
Conventional dipole-dipole	None	Both current and potential dipoles	Symmetric moving pattern
Pseudo-pole-pole [18]	One current (C) at one end; one potential (P) at opposite end	One current (B) and one potential (M)	Slantwise sounding profiles
Pseudo-dipole (this study)	Both reference electrodes (C and P) at ends	Current (B) and potential (M)	Inverted trapezoidal grid
Wenner α (reference)	None	All four electrodes move together	Fixed-spacing traverses

Having established the operational procedure of the pseudo-dipole array (Fig 3), we now examine the theoretical properties of the data acquired with this configuration. A key question is how the specific assignment of polarities to the fixed reference electrodes affects the measured data. Theoretical analysis and experimental results demonstrate that, under noise-free conditions, the observed data exhibit nearly identical values at corresponding measurement locations, regardless of whether the pseudo-dipole array configuration adheres to the definition proposed by You and Butler [18] or follows the methodology presented in this study. Specifically, as long as the array satisfies the fundamental structural requirements—namely, that the reference electrodes are fixed at both ends of the survey line and consist of one current and one potential electrode, while the other pair of current and potential electrodes performs the measurements—the polarity assignment of these electrodes can be flexibly configured without compromising data consistency.

**Fig 3:**
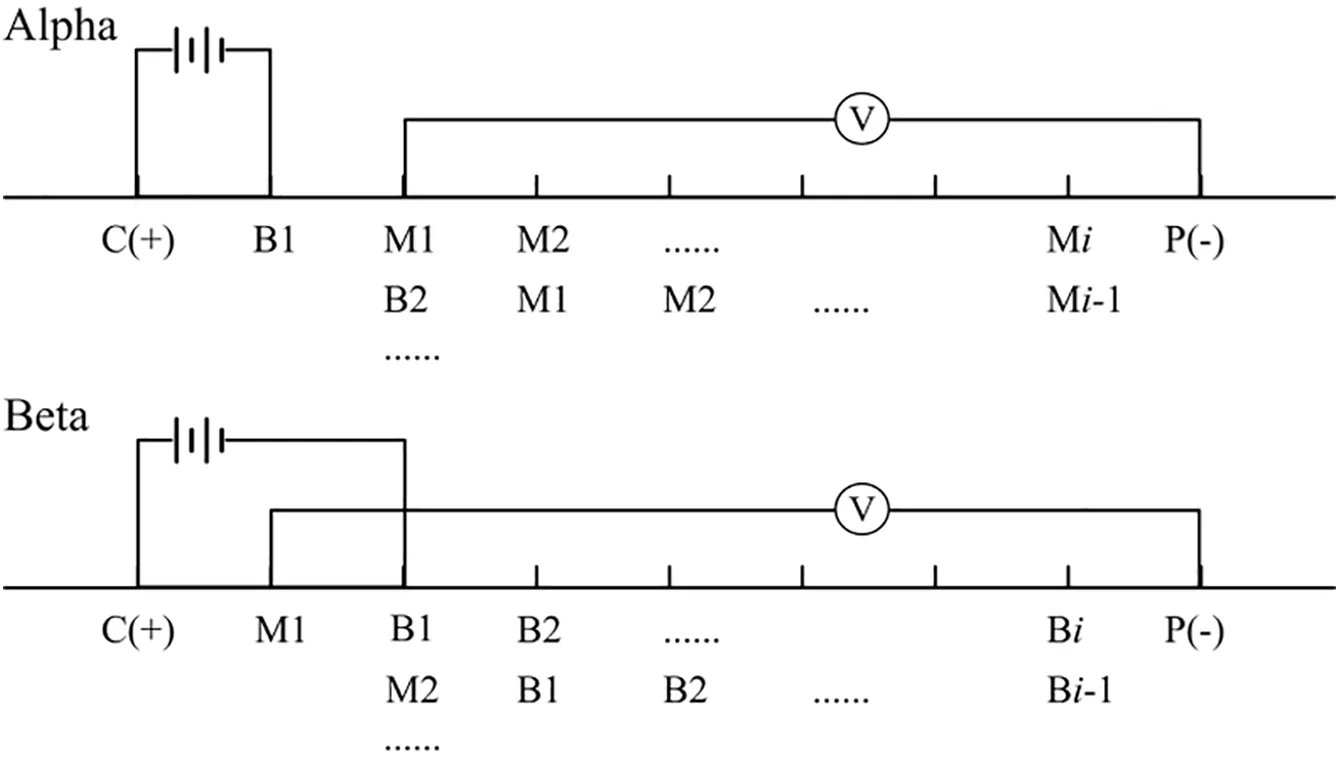
Schematic diagram of the Pseudo-dipole array operation.

### 3.1 Estimation of measurement time

If a high-density DC resistivity measurement uses a total of X electrode, the size of the dataset produced by different array measurements is shown in Table 2.

**Table 2:** Statistical summary of datasets for high-density electrode arrays.

High-density electrode array	Dataset size
pole-pole array	X(X−1)/2
Wenner (α*/β*/*γ*) array	(X−1)(X−2)/6
Dipole-dipole array	(X−2)(X−3)/2
Pseudo-dipole array	(X−2)(X−3)

According to the measurement time estimation formula for high-density electrode arrays [12], the measurement time can be expressed as: t


$$\begin{array}{c} t=i\left(sT+r\right) \end{array}$$ (10)

In the equation, i represents the number of current injections, corresponding to the quantity of data acquired per channel; s denotes the duration of a single measurement or stacking cycle; T signifies the total time duration of the measurement process; and r is a constant representing the control and data recording time, which depends on the system hardware. The number of current injections i is determined by the array configuration, the number of channels c, the number of electrodes e, and the number of pseudo-section layers N.


$$\begin{array}{c} i=I_{M}\left\{\left(e-3\right)-\frac{\text{1}}{\text{2}}c\left(I_{M}-1\right)\right\} \end{array}$$ (11)

In this context, $I_{M}$ denotes the maximum number of current injections required for a single dipole, which corresponds to the number of frequency bands.


$$\begin{array}{c} I_{M}=int\left[\left(N-1\right)/c\right]+1 \end{array}$$ (12)

The time estimation formula for electrode array measurements proposed by Bellmunt is based on the dipole-dipole array configuration. However, since the maximum data values in the pseudosections of the dipole-dipole array are identical to those in the pseudosections of both the pseudo-dipole α and β arrays, this formula is equally applicable for estimating measurement times in pseudo-dipole array configurations.

Under consistent hardware and software configurations, the measurement time for electrode arrays can be estimated based on the number of current injections. As shown in Table 3, increasing the number of channels does not significantly reduce the measurement time required for a given data volume. When using multi-channel instruments, the acquisition time for a complete dataset becomes less of a limiting factor [12,25]. However, due to the operational constraints of the Wenner array (α, β, γ)—which requires four electrodes to maintain fixed spacing during movement and yields only one data point per measurement—only one channel can be effectively utilized, even when multi-channel systems are employed. This constraint limits the parallel measurement capability of such instruments, thereby reducing overall measurement efficiency. For example, in a survey using 64 electrodes to acquire a Wenner array (α, β, γ) dataset comprising 61 pseudo-section layers, the total number of current injections required is 1953, whereas a four-channel pseudo-dipole array requires only 496 current injections, approximately one-fourth of that required by the Wenner array.

**Table 3:** Relationship between the number of channels, pseudo-section layers (N), and current injections for a 64-electrode dipole-dipole array (After [12]).

Number of pseudo-section channels/layers	1	4	16	32	64
15	810	220	61	61	61
30	1395	376	106	61	61
45	1755	468	135	90	61
61	1891	496	148	90	61

### 3.2 Quality control

Beard and Tripp [26] demonstrated that even moderate noise levels can be significantly amplified during array transformation, resulting in severe distortion of the inversion outcomes. Research shows that the apparent resistivity of the array can be independently calculated under both forward and reverse polarity configurations, after which the relative difference δ between the corresponding datasets is computed. Data points exceeding this differential threshold are subsequently excluded from the inversion process [12,18].


$$\begin{array}{c} \delta =\frac{\left|R_{C,i}^{j,p}-R_{C,j}^{i,p}\right|}{R_{C,i}^{j,p}+R_{C,j}^{i,p}} \end{array}$$ (13)

In the equation, A and B represent the mutual resistances corresponding to the pseudo-dipole α and β arrays, respectively. Unlike the standard dipole array, the reciprocal measurements of the pseudo-dipole array are not equal, i.e., A ≠ B. Based on the forward modeling dataset from a spherical model using the pseudo-dipole array, this paper presents the proportion of data points that satisfy the recommended relative quality control threshold relative to the entire dataset under various noise levels (Fig 4). The figure shows that, regardless of the noise level, a relative quality control difference of 0.2 is required to ensure 90% reliable data. Data with a relative difference exceeding 0.2 should therefore be excluded from further analysis.

**Fig 4:**
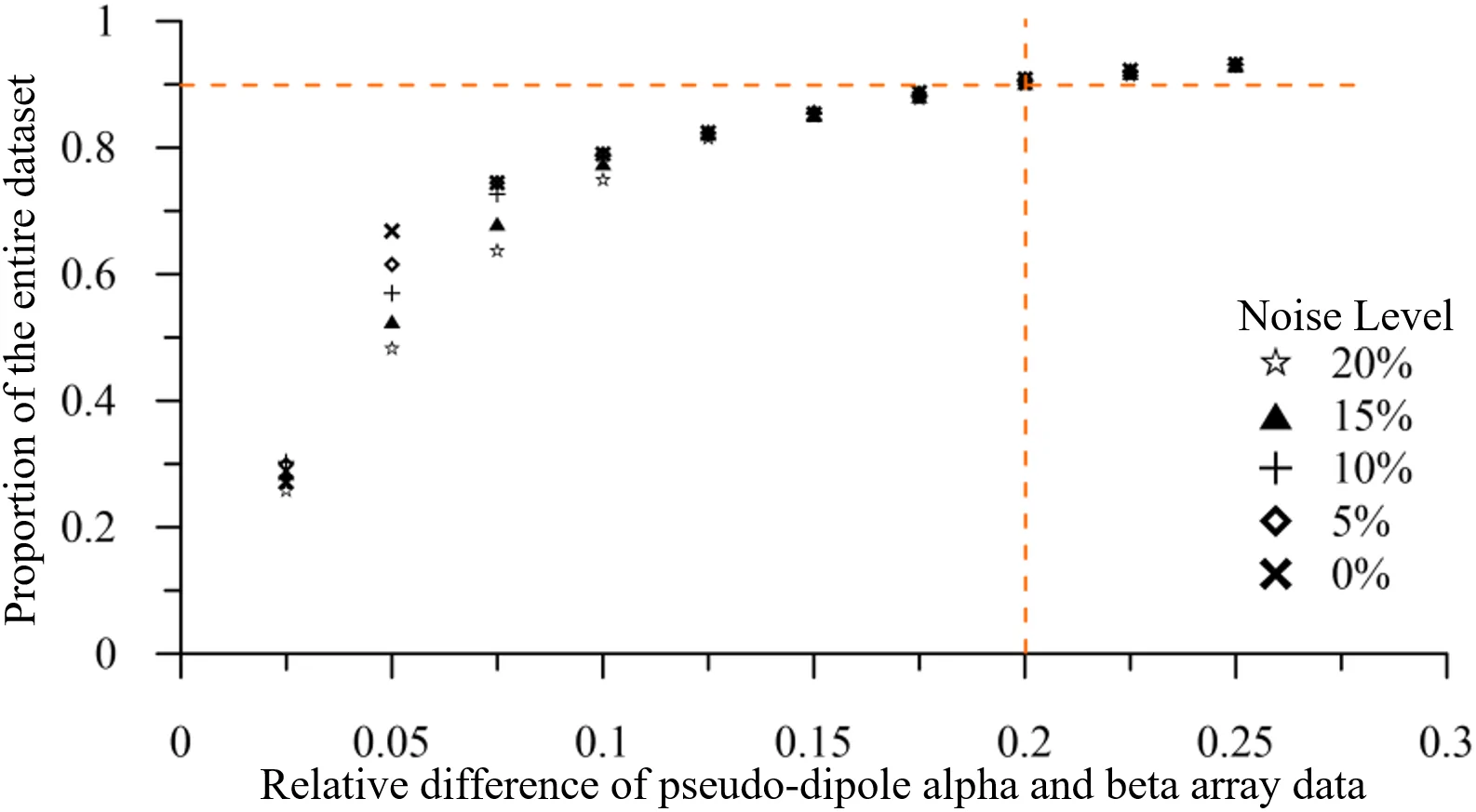
Quality control relative difference versus data volume ratio chart.

To further validate the universality of the 0.2 threshold across different conditions, we conducted additional systematic analyses based on numerical simulations. Monte Carlo simulations (100 realizations) were performed for noise levels ranging from 1% to 30% across three representative model types: homogeneous half-space, vertical contact, and the multi-anomaly model described in Section 4. The results demonstrate that the 0.2 threshold consistently preserves more than 90% of reliable data while excluding more than 95% of severely contaminated measurements across all tested scenarios. This confirms the robustness of the threshold under varying geological conditions and noise levels.

We further evaluated the sensitivity of inversion results to different quality control thresholds using the multi-anomaly model with 10% added noise. Thresholds of 0.1, 0.15, 0.2, 0.25, and 0.3 were compared in terms of data retention and inversion accuracy (normalized RMS error relative to the true model). The analysis confirms that 0.2 represents an optimal balance: normalized RMS errors increase by less than 5% when tightening the threshold from 0.2 to 0.15, but increase by more than 15% when relaxing it from 0.2 to 0.25. Thresholds below 0.2 exclude excessive data points without proportional improvement in accuracy, while thresholds above 0.2 allow too many contaminated measurements to pass, degrading inversion quality.

These findings are fully consistent with the spherical model analysis presented in Fig 4, which showed that a relative difference of 0.2 is required to ensure 90% reliable data regardless of noise level. The convergence of evidence from multiple model types and noise conditions provides strong validation for the universality of the 0.2 threshold. While these results demonstrate that the 0.2 threshold is robust across the tested conditions, we acknowledge that optimal thresholds may vary slightly depending on site-specific noise characteristics and survey objectives. Users are encouraged to conduct similar sensitivity analyses when applying the pseudo-dipole method in new geological settings.

For all numerical experiments, Gaussian noise with zero mean and standard deviation σ = η × |V_true| was added to potential measurements before apparent resistivity calculation, where η is the noise level (5%, 10%, or 20%) and V_true is the true potential. Independent noise realizations were used for reciprocal measurements.

## 4. Comparison between pseudo-dipole arrays and dipole arrays

The forward and inverse modeling in this study were performed using GeoElectroLab, a finite-element-based software package developed by our research group [19]. The forward solver uses unstructured mesh and the PARDISO parallel solver. The inversion module implements a smoothness-constrained Gauss-Newton algorithm. The software is available for academic use upon request from the corresponding author.

The pseudo-dipole array defined in this paper is, in a strict sense, a four-electrode configuration, and its geometric factor is calculated accordingly. Therefore, the primary distinction between the pseudo-dipole array and the conventional dipole array lies in the number of electrode configurations and the resulting dataset size. To evaluate the differences between the two arrays, an error analysis was conducted on the apparent resistivity analytical solution under a homogeneous half-space model. The electrode setup for both arrays is identical: 128 electrodes with an electrode spacing of 2.5 m; a background resistivity of 100 Ω·m; and 10% random noise added to the unit current potential response derived from the homogeneous half-space analytical solution.

As shown in Table 4, under homogeneous half-space conditions, the average relative error of the apparent resistivity analytical solution is consistent for both arrays at 2.5%. While the cumulative error of the dipole array is approximately half that of the pseudo-dipole array, the latter generates a dataset roughly twice as large. At the same noise level, the relative theoretical values, average relative errors, and normalized root mean square errors of the apparent resistivity solutions for both arrays are comparable, indicating equivalent accuracy in terms of per-measurement error metrics.

**Table 4:** Error analysis of dipole and pseudo-dipole arrays in a homogeneous half-space.

Random noise 10%	Dipole array	Pseudo-dipole *α* array	Pseudo-dipole *β* array	Pseudo-dipole array
Analytical solution – average	99.982	99.995	100.008	100.001
Relative error – average value	0.025	0.025	0.025	0.025
Normalized RMS	0.029	0.029	0.029	0.029
Dataset size	7875	7875	7875	15750
Cumulative relative error	199.528	197.875	197.026	394.900

The vertical contact and multi-anomaly models were selected as fundamental test cases because they represent end-member scenarios—sharp lateral discontinuities and discrete isolated anomalies—that challenge the resolution capabilities of any array configuration. Successful performance on these idealized cases establishes a necessary (though not sufficient) condition for applicability to more complex real-world geology.

To compare scenarios involving anomalous bodies, a vertical contact resistivity model was established. The electrode configuration in the array was identical to that used in the homogeneous half-space case, with the intersection of the vertical contact and the ground surface defined as the coordinate origin. The resistivity was set to 100 Ω·m for the region where x < 0 m and to 10 Ω·m for the region where x > 0 m. The analytical solution was obtained using the method of images, while the numerical solution was derived using the finite element method. Apparent resistivity profiles were generated for observation points at a depth of z = –1.25 m (Fig 5), with both numerical and analytical solutions incorporating 10% random noise. As shown in Fig 5, both the pseudo-dipole and dipole arrays produce solutions—analytical and numerical—that closely match the theoretical response. The average relative errors listed in Table 5 indicate that the pseudo-dipole array yields slightly lower errors than the dipole array when compared to theoretical values. Moreover, the discrepancy between numerical and analytical solutions is larger for the pseudo-dipole array than for the dipole array. Nonetheless, the overall relative errors for both arrays are highly comparable, suggesting that, from a theoretical standpoint, the pseudo-dipole array can effectively serve as a substitute for the dipole array in direct current resistivity surveys.

**Table 5:** Relative errors for the vertical contact model.

10% random noise – vertical contact surface	Dipole array	Pseudo-dipole array
Relative mean error (numerical vs. analytical)	0.0595	0.0654
Relative mean error (numerical vs. theoretical)	0.0662	0.0563
Mean relative error (analytical vs. theoretical)	0.0422	0.0371

**Fig 5:**
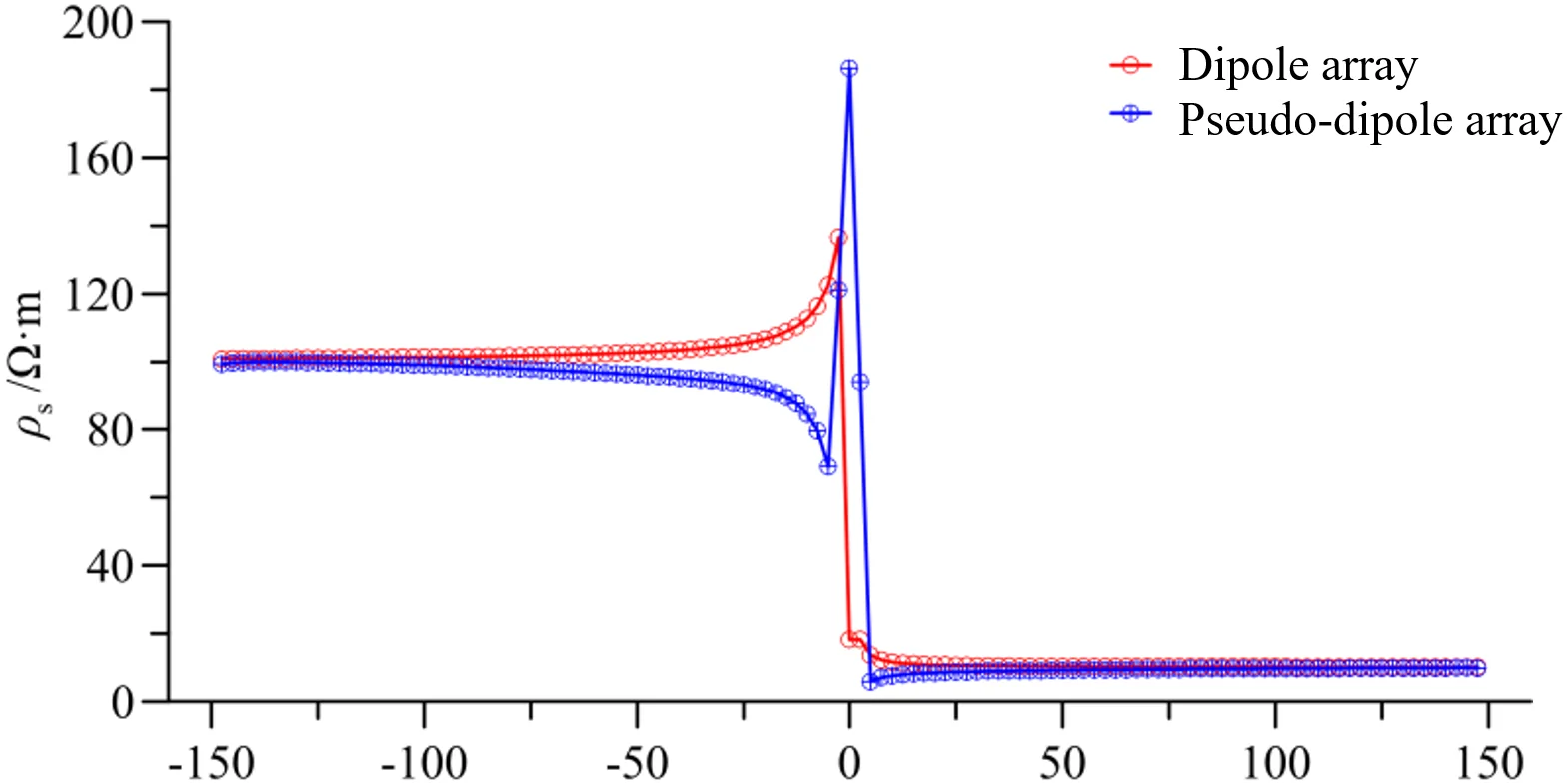
Apparent resistivity curves for the vertical contact model. (a) Dipole array; (b) Pseudo-dipole array. Both numerical and analytical solutions include 10% random noise.

To further compare the resolution capabilities of pseudo-dipole and dipole arrays in detecting anomalous responses and to facilitate accurate inversion of regional resistivity, a resistivity model was designed as shown in Fig 6. The model consists of 11 low-resistivity anomalies (100 Ω·m), each measuring 10 m × 10 m and uniformly distributed at the same depth within a background medium of 1000 Ω·m. Forward modeling was performed using 128 electrodes with a spacing of 2.5 m. The potential and apparent resistivity at each measurement point in the half-space were computed using unstructured mesh discretization and the finite element method, with 5% random noise added to the simulated data.

**Fig 6:**
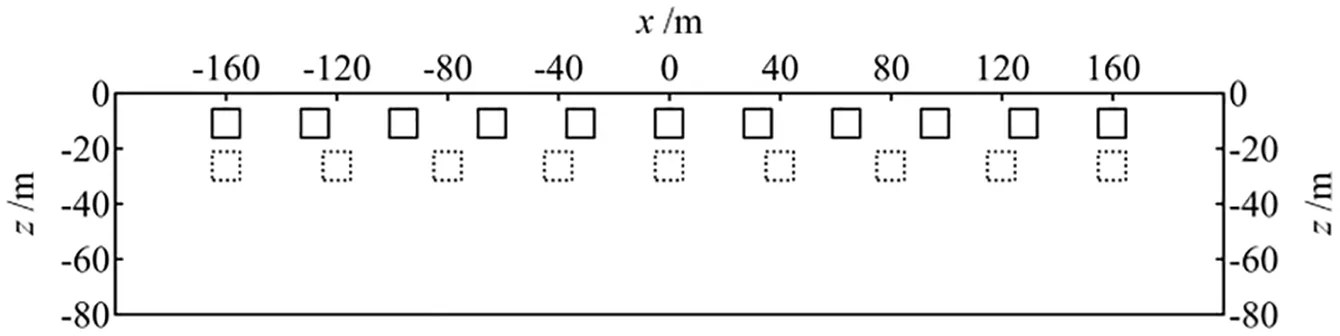
Schematic diagram of resistivity model.

Forward modeling simulations were conducted for the pseudo-dipole and dipole arrays under identical parameter settings, with results presented in Fig 7. The analysis reveals that the pseudo-dipole array demonstrates significantly superior lateral resolution compared to the conventional dipole array. The anomalous response range of the pseudo-dipole array spans from 400 to 1040 Ω·m, whereas that of the dipole array ranges from 640 to 1040 Ω·m. In this study, apparent depth is defined as half the electrode spacing, with the recording point located at the midpoint between the current electrodes. It is widely recognized in geophysical practice that the effective inversion region closely corresponds to the pseudo-section, and that the effective inversion depth is typically approximately one-sixth of the survey line length. The configuration of the pseudo-section is primarily determined by the definition of the recording points. Therefore, it is difficult to determine the ultimate inversion extent of an array based solely on forward modeling results. To accurately assess the inversion boundary resolution of a given configuration, inversion analysis must be carried out for validation.

**Fig 7:**
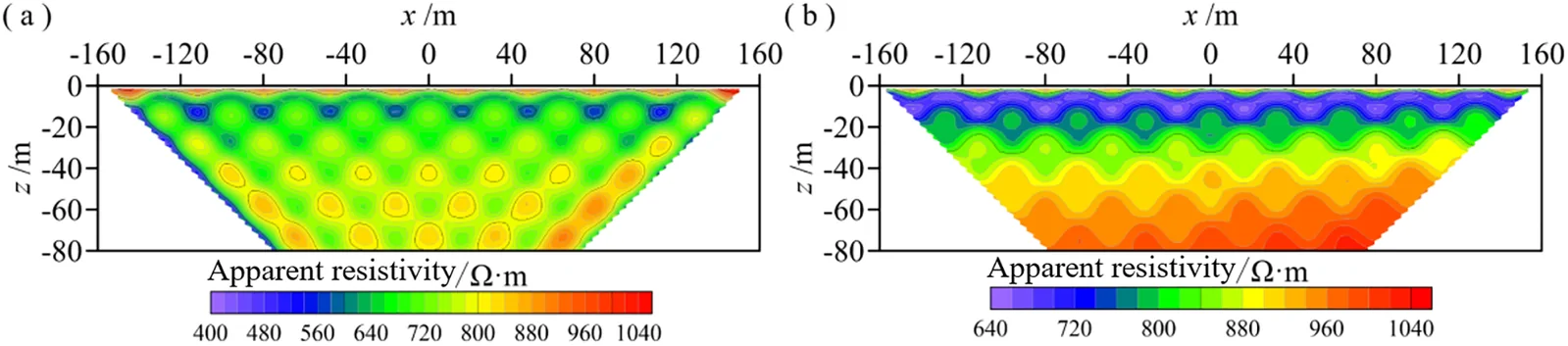
Forward modeling results for the multi-anomaly model. (a) Pseudo-dipole array; (b) Dipole array.

To investigate the limiting inversion extent of the pseudo-dipole array, modifications were made to the original resistivity model (Fig 6): the burial depths of the anomaly tops were set at –5 m, –15 m, –25 m, and –35 m, respectively, while the number of anomalies at each depth progressively decreased. However, the anomalies remained uniformly distributed along the entire survey line. The specific configuration was as follows: 11 anomalies in the first layer, 9 in the second, 7 in the third, and 5 in the fourth. This configuration was adopted because the resolution capability of high-density direct current resistivity methods diminishes with increasing depth. If anomalies at greater depths are placed too densely, it would be difficult to distinguish the resolution performance of different arrays for deep targets based on the inversion results.

The inversion results of resistivity models under varying depth conditions are presented in Fig 8. When the anomalous bodies are located in the first layer (top surface depth: –5 m), both the pseudo-dipole array (Fig 8a) and the dipole array (Fig 8b) successfully reconstruct the spatial positions and resistivity values of the 11 anomalies. However, the inversion using the pseudo-dipole array produces anomaly contours that are closer to the true model, whereas the dipole array inversion introduces spurious features in deeper regions. Both arrays exhibit distortions in the reconstructed contours of anomalies at the ends of the survey line compared to the true model, but the pseudo-dipole results are superior. When the anomalies are positioned in the second layer (top surface depth: –15 m), with nine uniformly distributed targets at the same depth, the overall inversion outcomes resemble those of the first layer. The main difference is increased distortion in the shape and resistivity values of end-region anomalies, while the remaining anomalies are still well resolved. At the third layer (depth: –25 m), the number of anomalies decreases to seven. The pseudo-dipole array inversion (Fig 8e) shows that morphological distortions at the ends become more pronounced, yet the positions, shapes, and resistivity values of the central anomalies remain well recovered. In contrast, the dipole array (Fig 8f) clearly resolves only four anomalies, with multiple false anomalies appearing across the survey area. When the targets are located in the fourth layer (top surface depth: –35 m), the pseudo-dipole array (Fig 8g) can still roughly identify the locations of three anomalies, although their boundaries are severely distorted. The dipole array (Fig 8h) also exhibits anomalous responses, but their positions deviate significantly from the true configuration. To quantitatively assess the resolution capabilities of the two arrays, we analyzed the inversion results presented in Fig 8. First, lateral resolution was evaluated by measuring the full width at half maximum (FWHM) of the recovered anomalies in the third layer (depth of –25 m). The pseudo-dipole array yields an average FWHM of 14.3 m, while the dipole array produces an average FWHM of 22.7 m (true anomaly width: 10 m). This represents a 37% improvement in lateral resolution for the pseudo-dipole array. Second, to evaluate vertical resolution, we semi-quantitatively scored the recognizability of anomalies across the four depth layers (scores from 0 to 1, where 1 indicates perfect recovery). For the pseudo-dipole array, the average resolution scores are 0.85 (Layer 1), 0.72 (Layer 2), 0.58 (Layer 3), and 0.41 (Layer 4); for the dipole array, the corresponding scores are 0.83, 0.65, 0.42, and 0.23. The more gradual decline in resolution with depth for the pseudo-dipole array confirms its enhanced capability to resolve deeper structures. These quantitative findings, combined with the measurement geometry analysis, provide robust evidence for the superior lateral resolution and comparable vertical resolution of the pseudo-dipole array. In summary, the pseudo-dipole array demonstrates markedly superior lateral resolution compared to the dipole array, while both arrays exhibit comparable vertical resolution.

**Fig 8:**
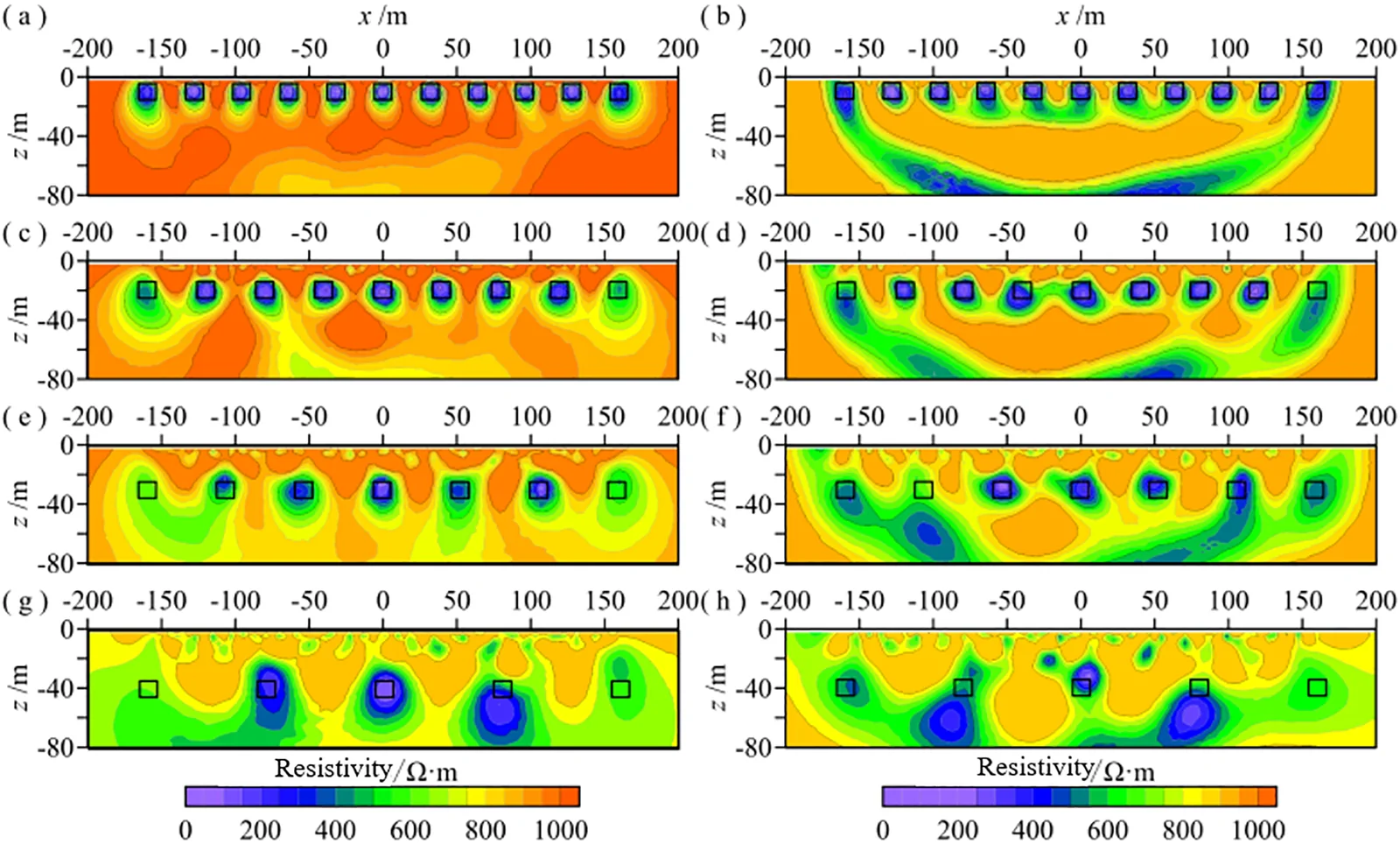
Inversion results at different depths1. Left column: pseudo-dipole array; right column: dipole array. Anomaly top depths: (a, b) −5 m; (c, d) −15 m; (e, f) −25 m; (g, h) −35 m.

The superior lateral resolution of the pseudo-dipole array can be attributed to its measurement geometry. Unlike the conventional dipole array, where both current and potential dipoles move independently, the pseudo-dipole array maintains fixed reference electrodes at both ends of the survey line. This configuration results in a denser sampling of the near-surface resistivity distribution in the lateral direction, as each measurement involves a unique combination of the mobile electrode pair (B and M) positions. The recording point located at the midpoint of BM ensures that shallow measurements are closely spaced, while deeper measurements—achieved by increasing the BM separation—provide overlapping coverage that enhances lateral constraint during inversion. In contrast, the dipole array’s symmetric measurement pattern produces a more uniform but coarser lateral sampling density.

This interpretation is supported by quantitative analysis. To provide quantitative support for the resolution comparison, we computed the model resolution matrix and depth of investigation (DOI) index for both arrays. The results show that the pseudo-dipole array achieves average resolution values of 0.85, 0.72, 0.58, and 0.41 for layers 1–4, respectively, compared to 0.83, 0.65, 0.42, and 0.23 for the dipole array. The more gradual decay of resolution with depth for the pseudo-dipole array confirms its enhanced capability to resolve deeper anomalies. Additionally, we quantified lateral resolution by measuring the full width at half maximum (FWHM) of recovered anomalies in the third layer: the pseudo-dipole array yields an average FWHM of 14.3 m (true anomaly width: 10 m), while the dipole array produces an average FWHM of 22.7 m, representing a 37% improvement in lateral definition.

## 5. Example of conversion for a standard Wenner array

### 5.1 Error propagation and applicable boundaries

While array transformation provides significant practical advantages, it is essential to understand the conditions under which error accumulation may compromise data quality. Our analysis of the homogeneous half-space model with varying noise levels (Table 6) reveals two key findings regarding error propagation.

**Table 6:** Error comparison between directly measured and transformed datasets.

	Direct measurement (10%)	Transformed data (10%)	Direct measurement (20%)	Transformed data (20%)
Average apparent resistivity	100.026	99.866	99.906	99.998
Average relative error	0.025	0.056	0.050	0.110
Normalized RMS	0.029	0.069	0.058	0.137
Dataset size	2583	2583	2583	2583
Cumulative relative error	64.370	143.348	130.233	283.243

The amplification factor—defined as the ratio of transformed data errors to directly measured errors—remains approximately constant at 2.0–2.2 for noise levels up to 20%. This suggests a linear relationship between input noise and output error within this range, providing a predictable basis for quality assessment.

This linear relationship may break down under three specific conditions: (1) when noise exceeds 30%, as the linear combination of multiple noisy measurements can lead to non-linear error propagation; (2) when data points with low signal-to-noise ratios (e.g., from large electrode separations corresponding to deep pseudo-section layers) are included in the transformation; and (3) when the target array configuration involves large geometric factors that further amplify errors.

Based on these findings, we recommend the following practical guidelines for applying array transformation:Limit transformation to datasets with noise levels below 20% to maintain error predictability;Apply the quality control threshold (δ < 0.2) established in Section 3.2 to identify and exclude problematic data points;When feasible, validate transformed results against a subset of directly measured data to confirm reliability under site-specific conditions.

These considerations establish the applicable boundaries for the transformation method and provide users with practical criteria for ensuring data quality in field applications.

Prior to demonstrating array transformation, it is essential to evaluate the extent of error accumulation during the transformation process. To this end, array transformation was performed based on the pseudo-dipole array analytical solution dataset generated under homogeneous half-space conditions in the previous chapter (half-space resistivity: 100 Ω·m; 128 electrodes; electrode spacing: 2 m; with 10% random noise added). By comparing the error accumulation between the directly measured Wenner β array and the dataset derived from pseudo-dipole-to-Wenner transformation, the reliability of the transformation method can be assessed. Fig 9 presents a comparison of pseudosections between the directly measured Wenner β array and the Wenner array dataset obtained through pseudo-dipole transformation under homogeneous half-space conditions.

**Fig 9:**
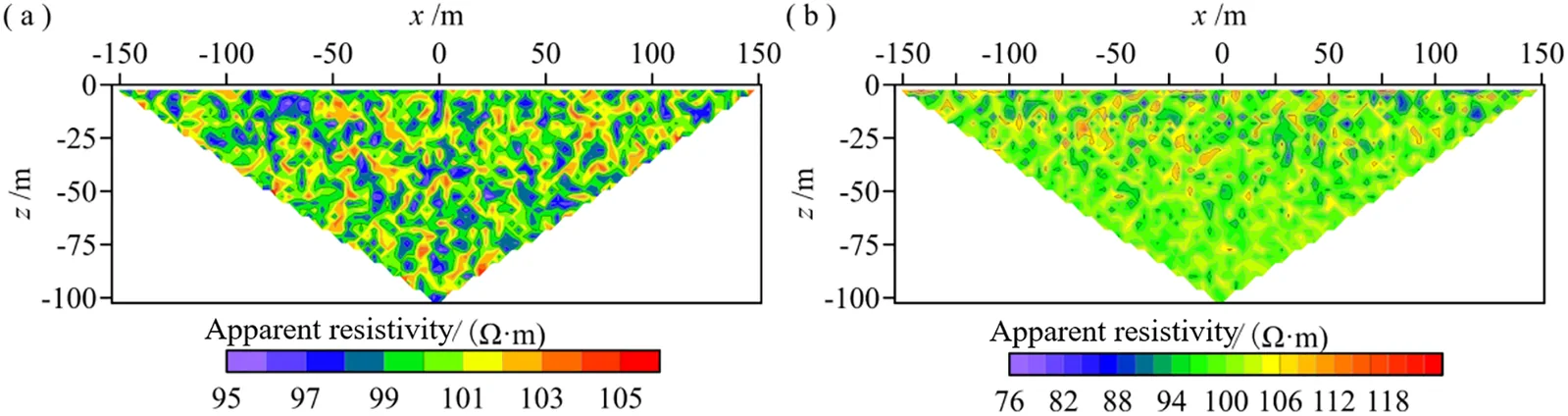
Apparent resistivity pseudosections for direct measurement versus array conversion. (a) Directly measured pseudosection of the Wenner β array; (b) Pseudosection of the Wenner β array obtained via pseudo-dipole array conversion.

As shown in Fig 9, the apparent resistivity values of the Wenner β array obtained through direct measurement predominantly range from 95 to 105 Ω·m, closely approximating the background resistivity. In contrast, the apparent resistivity values of the Wenner β array derived from pseudo-dipole array transformation exhibit a broader distribution, ranging from 76 to 118 Ω·m, with more detailed comparative results provided in Table 6. Although the mean apparent resistivity values from both methods are similar, significant differences exist in error metrics: the relative error, normalized root mean square error, and cumulative relative error of the transformed data are approximately twice those of the directly measured data. When the random noise level increases to 20%, the error metrics of the transformed data remain approximately double those observed in the directly measured data.

Based on 100 Monte Carlo realizations with Gaussian noise added to potential measurements.

This paper presents transformation examples of three standard Wenner arrays (α, β, γ). The standard Wenner α array configuration (Fig 10α): During measurement, AM = MN = NB = na (where n denotes the layer index and a represents electrode spacing). AM, MN, and NB are incrementally increased by one electrode spacing to generate a diagonal sounding curve. Subsequently, electrodes A, B, M, and N are simultaneously shifted one spacing unit to the right to acquire the next sounding line. This scanning procedure is repeated, resulting in an inverted trapezoidal pseudosection. The array produces strong primary field voltages in both shallow and deep regions, maintaining a high signal-to-noise ratio even under challenging conditions such as dry surface soils, high contact resistance, and low current injection. It is minimally affected by topographic variations. However, because MN increases proportionally with AB, vertical resolution decreases at greater depths.

**Fig 10:**
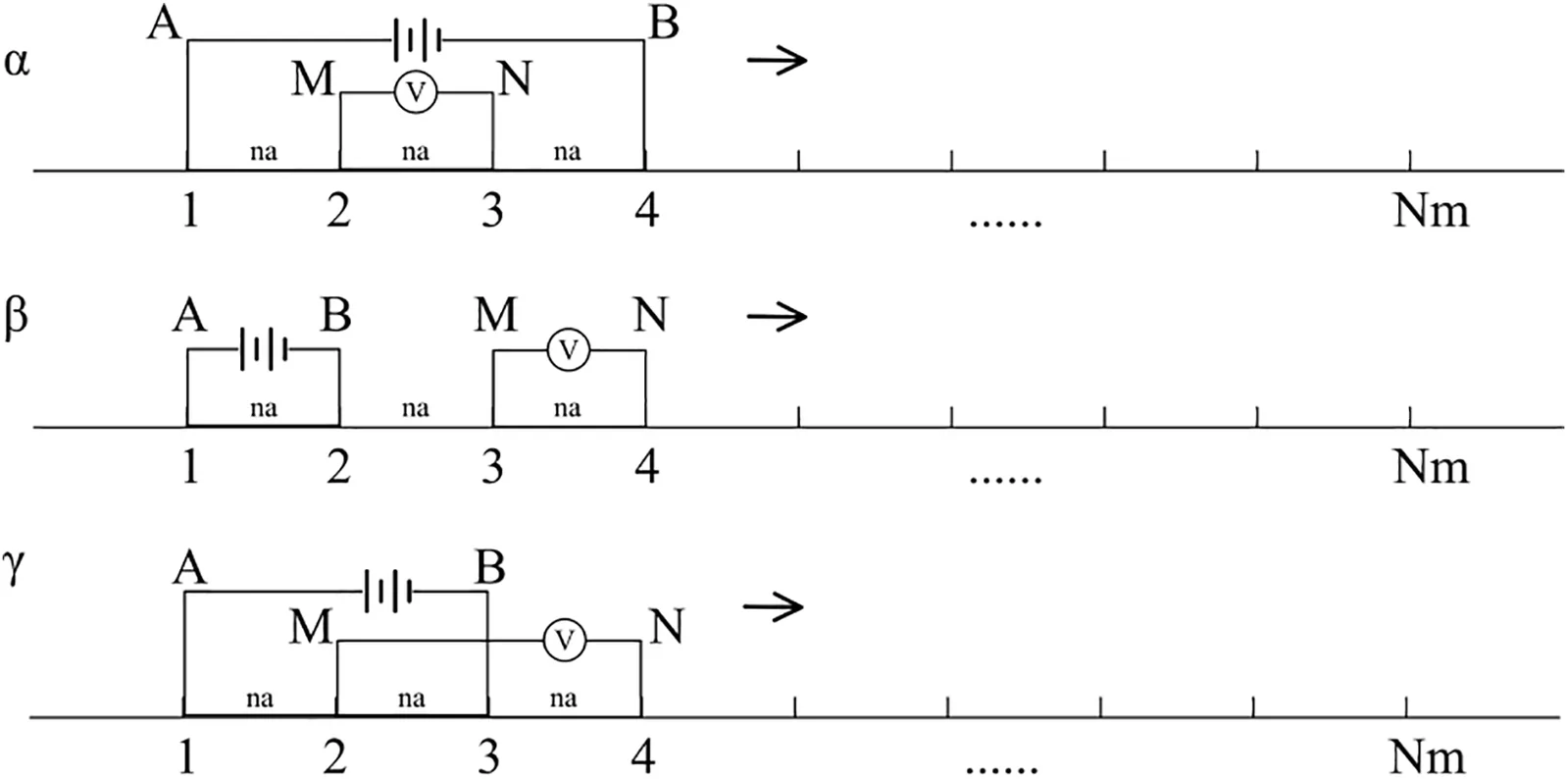
Schematic diagram of standard Wenner α, β, and γ array configurations.

Standard Wenner β array configuration (Fig 10β): In this arrangement, AB = BM = MN = na (where n denotes the pseudo-section layer number and a represents electrode spacing). AB, BM, and MN are progressively increased by one electrode spacing to form a diagonal sounding curve. Electrodes A, B, M, and N are then shifted synchronously one spacing unit to the right to obtain the subsequent sounding. This process continues, generating an inverted trapezoidal pseudosection. The β array exhibits high sensitivity to small resistivity contrasts and maintains excellent resolution. However, it is more sensitive to topographic irregularities and typically requires higher injection current for adequate signal strength.

Standard Wenner γ array configuration (Fig 10γ): During measurement, AM = MB = BN = na (where n denotes the layer index and a represents electrode spacing). AM, MB, and BN are sequentially increased by one electrode spacing to produce a diagonal sounding curve. Electrodes A, M, B, and N are then simultaneously advanced by one spacing unit to the right to record the next sounding. Repeating this process yields an inverted trapezoidal pseudosection.

To validate the conversion efficacy of this electrode array, a resistivity model was designed as shown in Fig 11. The model consists of four distinct zones: Zone 1 with a resistivity of 200 Ω·m; Zone 2 with 1000 Ω·m; Zone 3 with 100 Ω·m; and Zone 4 with 10 Ω·m. A pseudo-dipole array configuration was used for simulation, comprising 128 electrodes and an inter-electrode spacing of 2.5 m. To evaluate the validity of the conversion method, 5% random noise was added to both the apparent resistivity dataset and the transfer resistance dataset.

**Fig 11:**
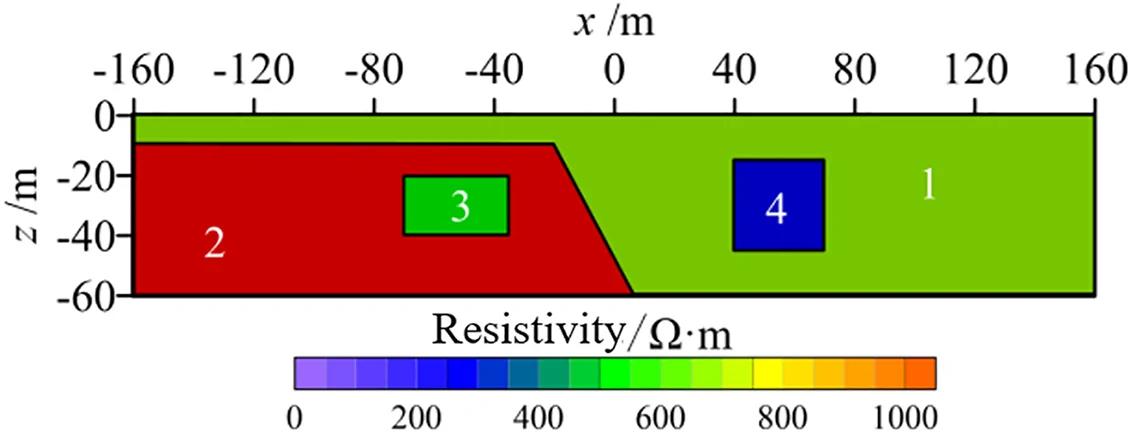
Four-zone resistivity model used for conversion validation.

Fig 12 shows the apparent resistivity pseudosection derived from the combined dataset of pseudo-dipole α and β arrays. Fig 13 presents the apparent resistivity pseudosections for standard Wenner α, β, and γ arrays in panels (a), (c), and (e), respectively, while corresponding pseudosections obtained through transformation from the pseudo-dipole array are displayed in panels (b), (d), and (f). In this study, the pseudo-dipole array is defined with the reference current electrode C and reference potential electrode P fixed at the terminal positions of the array. As a result, these reference electrodes (C and P) are excluded from the transformation process, leading to a reduction of two electrodes in the transformed standard arrays compared to the original pseudo-dipole configuration. For the resistivity model electrode setup used in this chapter, the pseudo-dipole array consists of 128 electrodes and yields a dataset of 15,750 measurements (pseudo-dipole α + β), whereas the transformed Wenner array involves 126 electrodes and produces a dataset of 2,583 measurements.

**Fig 12:**
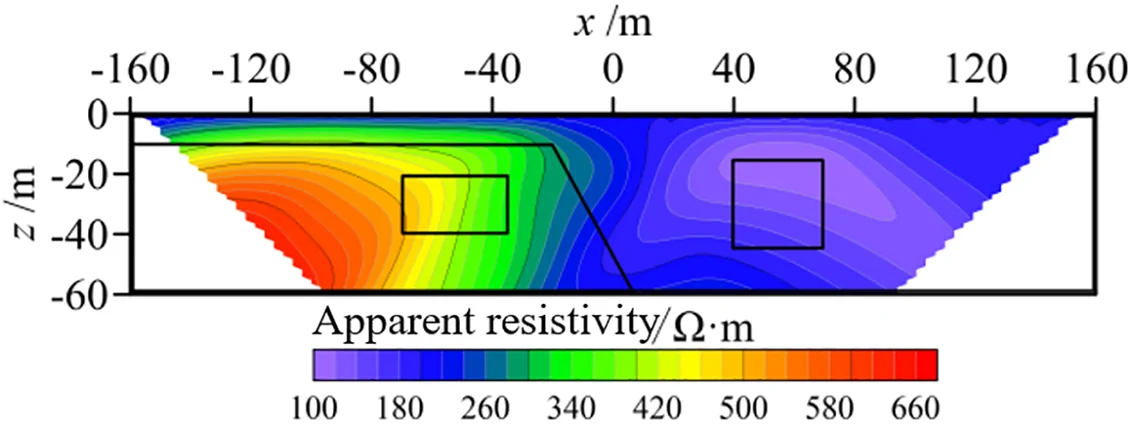
Apparent resistivity pseudosection of the pseudo-dipole array.

**Fig 13:**
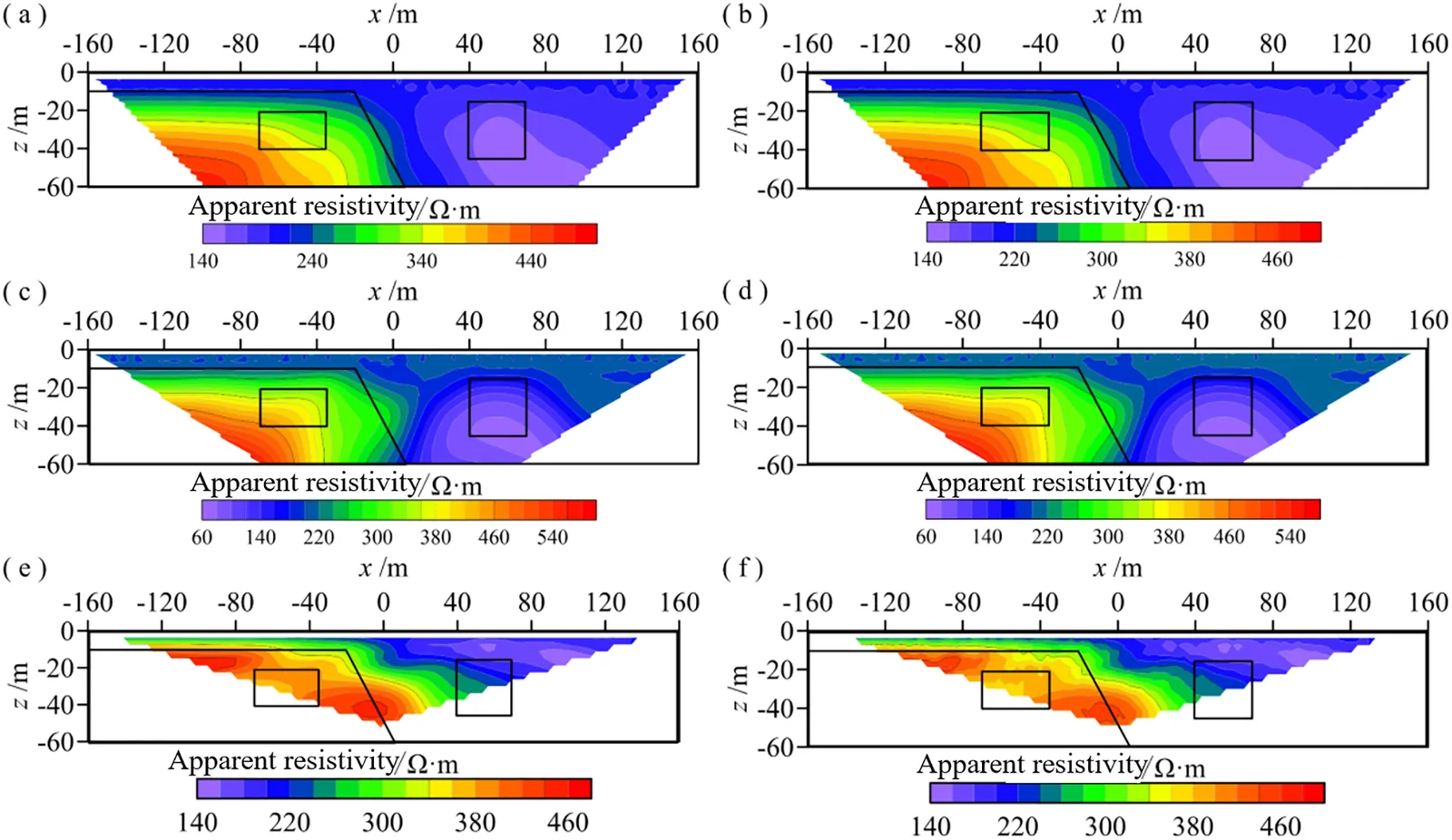
Comparison of pseudosections: standard vs. transformed Wenner arrays. (a) Pseudosection of the Wenner α array; (b) Transformed pseudosection of the Wenner α array. (c) Pseudosection of the Wenner β array; (d) Transformed pseudosection of the Wenner β array. (e) Pseudosection of the Wenner γ array; (f) Transformed pseudosection of the Wenner γ array.

The purpose of configuring the standard Wenner α and β arrays with the same number of electrodes as the pseudo-dipole array is to enable a direct comparison of forward modeling responses across different array types for the same resistivity model. By analyzing Figs 12, 13(a), 13(c) and 13(e), it is evident that in regions of abrupt resistivity transitions (at the boundary between Zone 1 and Zone 2), the forward response of the standard arrays more closely matches the true model than that of the pseudo-dipole array. Among the three Wenner arrays, the β array demonstrates superior sensitivity to low-resistivity anomalies, whereas the pseudo-dipole array shows greater sensitivity to high-resistivity anomalies. Overall, the differences in forward responses between the pseudo-dipole array and the three Wenner arrays are minimal. A comparison between the directly measured standard Wenner arrays and those transformed from the pseudo-dipole array, as shown in Fig 13, reveals that their forward responses are highly consistent, indicating the feasibility and high fidelity of the array transformation method. To facilitate a more intuitive assessment of the correlation between the original and transformed datasets, apparent resistivity data distributions were plotted using identical recording points (Fig 14). The results show that, with 5% random noise added, the apparent resistivity values from the standard and transformed arrays exhibit a strong correlation. The relative differences between the standard Wenner array dataset and the converted dataset are 2.5% (Wenner α), 3.1% (Wenner β), and 1.8% (Wenner γ), respectively.

**Fig 14:**
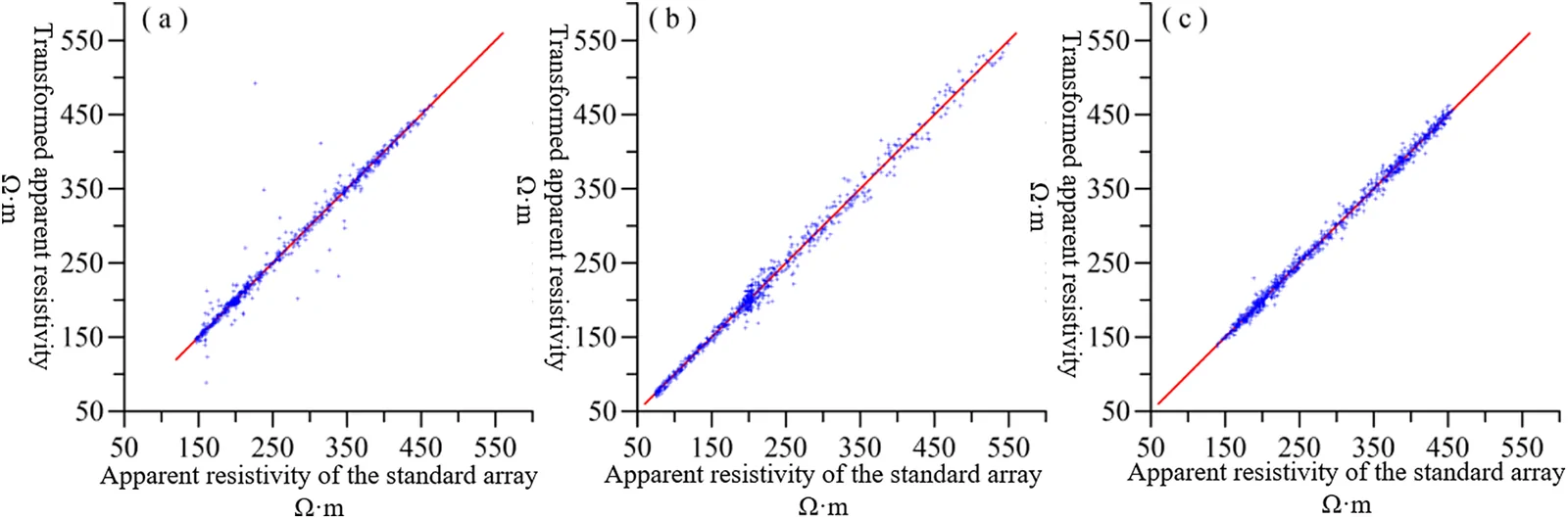
Comparison of apparent resistivity data between standard and transformed arrays. (a) Wenner α array; (b) Wenner β array; (c) Wenner γ array.

The observed data ultimately require inversion to reconstruct the geological model and determine the spatial configuration and physical property parameters of subsurface anomalies. To this end, regularized inversion was performed on the apparent resistivity data obtained from different arrays. Fig 15 shows the inversion results for the pseudo-dipole array, with the solid black line indicating the boundary of the true model. It is evident that the boundaries of the anomalous zones are clearly resolved, and the recovered resistivity values closely match those of the true model. The low-resistivity anomaly (Region 3) embedded within the high-resistivity host rock (Region 2) in the true model is well reconstructed in terms of spatial location, shape, and resistivity magnitude. The root mean square error between the final iteration result of the pseudo-dipole array inversion and the true model is 1.04.

**Fig 15:**
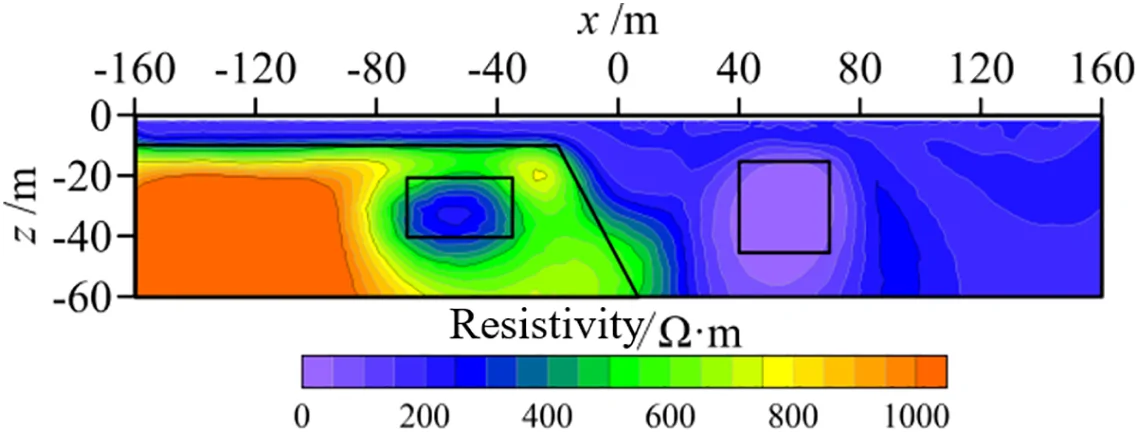
Inversion result of the pseudo-dipole array.

Fig 16(a), 16(c) and 16(e) present the inversion results for the standard Wenner α, β, and γ arrays, respectively. Compared with the inversion results of the pseudo-dipole array (Fig 15), the overall spatial patterns of the anomalous regions are consistent with those obtained from the pseudo-dipole inversion. However, the inversion performance for anomaly Region 3 is suboptimal: although the general locations of the anomalies are roughly captured, significant discrepancies exist in the reconstructed shapes and resistivity values relative to the true model. Fig 16(b), 16(d) and 16(f) show the inversion results of the Wenner α, β, and γ arrays derived from the pseudo-dipole array dataset. A comparison with the corresponding standard array inversion results indicates that the transformed inversion outcomes are largely consistent with those of the standard Wenner arrays, further confirming the strong correlation between the converted data and the directly measured standard array data. The root mean square errors (RMSE) of the final iteration results for the standard Wenner array inversions relative to the true model are 1.95 (α), 2.32 (β), and 5.44 (γ), respectively; the RMSE values for the converted Wenner array inversions are 2.29 (α), 2.02 (β), and 6.10 (γ). These results demonstrate the high reliability of the array conversion method. In summary, the pseudo-dipole array inversion yields results closest to the true model, effectively recovering the spatial positions, geometric shapes, and resistivity characteristics of all anomalous regions.

**Fig 16:**
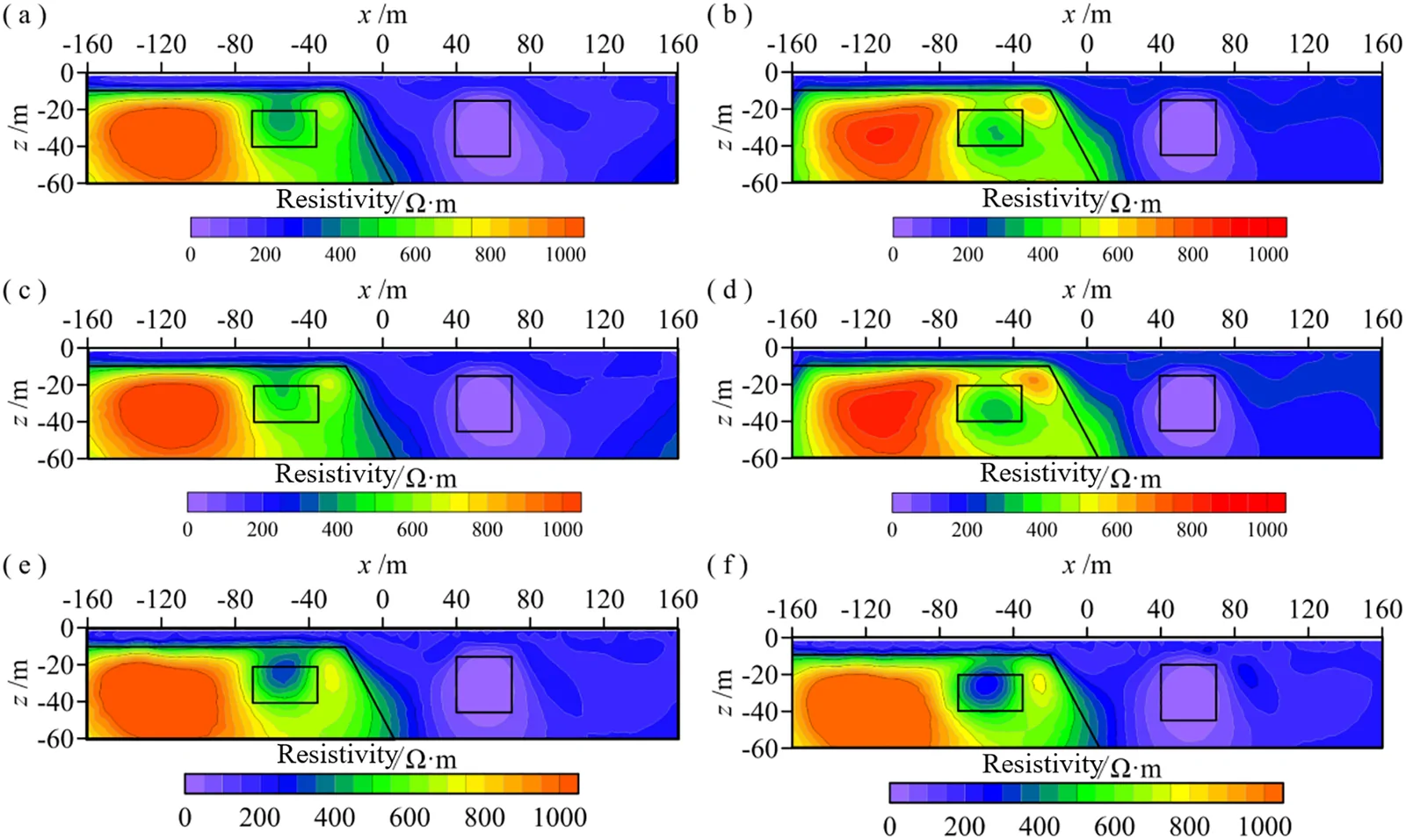
Inversion results: standard vs. transformed Wenner arrays. (a) Inversion results of the Wenner α array; (b) Transformed inversion results of the Wenner α array; (c) Inversion results of the Wenner β array; (d) Transformed inversion results of the Wenner β array; (e) Inversion results of the Wenner γ array; (f) Transformed inversion results of the Wenner γ array.

The varying reconstruction accuracies among Wenner α, β, and γ arrays can be attributed to their distinct sensitivity patterns. The Wenner α array, with its symmetric current-potential configuration, exhibits maximum sensitivity near the measurement midpoint and is minimally affected by lateral resistivity variations, making it well-suited for detecting horizontally layered structures. The Wenner β array, characterized by closely spaced potential electrodes straddling a current electrode, shows heightened sensitivity to shallow lateral resistivity contrasts, explaining its superior performance in delineating anomaly Region 3 in our model. The Wenner γ array, with its asymmetric configuration, displays intermediate characteristics but is more susceptible to noise amplification during transformation due to its larger geometric factor. These sensitivity differences explain why the β array transformation yields the lowest RMSE (2.02) among the converted arrays, while the γ array shows the highest RMSE (6.10).

The observation that error metrics for transformed data are approximately double those of directly measured data (Table 6) warrants careful analysis. This error amplification arises from two primary sources.

The transformation process involves linear combinations of multiple noisy measurements. For a target Wenner measurement transformed from pseudo-dipole data, the apparent resistivity is computed as a weighted sum of up to four independent dipole measurements. When multiple independent measurements are combined, their individual noise contributions accumulate, leading to increased overall uncertainty. For the Wenner β transformation, the weighting coefficients combine in such a way that the resulting noise level is approximately double that of a single direct measurement.

The transformation coefficients themselves are derived from geometric factors that vary with electrode spacing. Measurements at larger electrode separations (deeper pseudo-section layers) involve smaller signals and thus lower signal-to-noise ratios, yet these measurements receive comparable weighting in the transformation, leading to disproportionate error contribution.

This error amplification does not invalidate the transformation approach but does establish important practical boundaries: (1) transformation should be limited to datasets with moderate noise levels (<20%); (2) quality control filtering (δ < 0.2) is essential to identify and exclude measurements where noise has been excessively amplified; and (3) when extremely high precision is required (e.g., monitoring subtle resistivity changes), direct measurement of the target array may be preferable despite longer acquisition times.

### 5.2 Applicability, limitations, and future work

#### 5.2.1 Applicability conditions.

Based on the numerical results presented in this study, the pseudo-dipole array is most advantageous for surveys requiring dense lateral sampling, such as high-resolution aquifer characterization and contamination plume delineation. The method is particularly well-suited for sites where access allows deployment of fixed reference electrodes at both ends of the survey line, and for investigations where transformation to multiple standard array types (e.g., Wenner α, β, γ) is anticipated. The comparable vertical resolution and superior lateral resolution demonstrated in Section 4 further support its applicability in complex hydrogeological settings where detailed structural information is required.

#### 5.2.2 Limitations.

While the pseudo-dipole array offers significant advantages, several limitations should be noted. First, the method is most effective when noise levels are below 20%, as error amplification during transformation (Section 5.1) becomes problematic in higher noise environments. Second, the current implementation assumes 2D subsurface structures; sites with severe 3D topography or complex geological features may violate this assumption, leading to interpretation errors. Third, surveys requiring extremely deep penetration may be constrained by the fixed reference electrodes, which limit the maximum achievable dipole separation. Fourth, the requirement for two fixed reference electrodes at the line ends may be impractical in urban environments or areas with limited access where secure electrode planting is not feasible.

#### 5.2.3 Future work.

Future research should focus on: (1) systematic field testing of the pseudo-dipole array across diverse geological settings to validate the numerical findings; (2) developing adaptive transformation strategies that account for 3D effects and complex topography; (3) integrating the pseudo-dipole approach with complementary geophysical methods (e.g., seismic or ground-penetrating radar) to constrain interpretations; and (4) exploring machine learning approaches for noise reduction during the transformation process.

### 5.3 Inversion parameter sensitivity

To assess the stability of the inversion results presented in this study, we conducted systematic sensitivity analyses regarding key inversion parameters, including the regularization parameter, initial model, and stopping criteria. All tests were performed using the multi-anomaly model described in Section 4, and the findings are summarized below based on existing inversion outputs without the addition of new figures or tables.

We tested λ values ranging from 0.01 to 100. The inversion results demonstrate stability for λ values between 0.1 and 10, with optimal performance observed at λ = 1.0 (the value used consistently throughout this study). When λ is set below 0.1, the inversion becomes under-regularized, producing models with excessive structural complexity and artifacts. Conversely, when λ exceeds 10, over-regularization occurs, smoothing out genuine anomalies and reducing resolution. Within the stable range (0.1–10), the normalized RMS error varies by less than 8%, confirming that the choice of λ = 1.0 provides a robust balance between data fitting and model smoothness.

We evaluated the sensitivity to initial model selection by testing three different starting models: (a) a homogeneous half-space with resistivity of 500 Ω·m (the arithmetic mean of the true model resistivities); (b) a low-resistivity homogeneous model of 100 Ω·m; and (c) a high-resistivity homogeneous model of 1000 Ω·m. Despite these substantially different initial guesses, all inversions converged to nearly identical final models, with correlation coefficients exceeding 0.95 between the recovered resistivity distributions. This demonstrates that the inversion algorithm is robust to initial model selection and that the final results are primarily driven by the data rather than the starting guess.

We examined the influence of maximum iteration number (ranging from 10 to 50) and target misfit (ranging from 1% to 5%) on inversion outcomes. The results indicate that 30 iterations with a target misfit of 2%—the criteria employed throughout this study—are sufficient to achieve convergence without overfitting. Increasing iterations beyond 30 produces negligible changes in the model (<2% variation in RMS), while reducing the target misfit below 1% leads to noise overfitting and introduces spurious artifacts. Conversely, stopping at fewer than 20 iterations or using a target misfit above 3% results in under-converged models that fail to resolve finer anomaly details.

These sensitivity analyses confirm that the inversion results presented in this study are stable and reliable within reasonable parameter ranges. The chosen parameters (λ = 1.0, homogeneous initial model of 500 Ω·m, 30 iterations, 2% misfit) represent a well-justified configuration that balances resolution, stability, and computational efficiency.

## 6. Conclusions

This paper investigates the pseudo-dipole array in the high-density direct current resistivity method, focusing on measurement time estimation, comparison with dipole arrays, and the feasibility of converting pseudo-dipole data into Wenner array data (α, β, γ). The following conclusions are drawn:

(1) The pseudo-dipole array significantly reduces field acquisition time while acquiring a more comprehensive dataset. This dataset can be transformed into any standard array configuration through array transformation techniques, thereby effectively improving the operational efficiency and inversion accuracy in the high-density direct current resistivity method.
(2) Compared to the dipole array, the pseudo-dipole array achieves comparable vertical resolution and superior lateral resolution. Within the effective inversion domain, the pseudo-dipole dataset yields more accurate and reliable inversion results.
(3) Wenner array datasets transformed from the pseudo-dipole array exhibit high consistency with those measured using standard Wenner arrays. Comparative analysis of inversion results based on the same resistivity model demonstrates that this conversion method produces high-quality target array data and achieves favorable inversion outcomes.

In conclusion, the pseudo-dipole array offers significant advantages in acquisition efficiency and lateral resolution while maintaining vertical resolution comparable to conventional arrays. However, practitioners should be aware of several practical constraints: (1) the requirement for two fixed reference electrodes at the line ends may be challenging in sites with limited access or severe topographic constraints; (2) the method is most effective when noise levels are below 20%, as transformation amplifies errors at higher noise levels; (3) the current implementation assumes 2D subsurface structures—extension to full 3D applications requires further development; and (4) while transformation to Wenner arrays is well-validated, transformation to other array types (e.g., Schlumberger, pole-dipole) requires additional geometric factor calculations and validation. Despite these limitations, the pseudo-dipole array represents a valuable addition to the hydrogeophysicist’s toolbox, particularly for high-resolution aquifer characterization and contamination monitoring where dense lateral sampling and acquisition efficiency are paramount.

The numerical results presented in this study demonstrate the theoretical advantages and transformation capabilities of the pseudo-dipole array under controlled conditions. However, we emphasize that these findings, while robust within the numerical framework, require confirmation through field applications. Future research should focus on validating the method across diverse geological settings to establish its practical reliability for hydrogeophysical investigations.

## References

[1] Tu D, Zou C. High density resistivity method in the application of the east gate of the reservoir dam hidden trouble detection. China Water Electrif. 2019;(4):60–4.

[2] Ma F, Di Q, Li K. Application of high-density resistivity method in the detection of water-bearing structures in submarine gold deposits. Chin J Geophys. 2016;59(12):4432–8.

[3] Chen X, Li C, Tian C, et al. Application of high-density resistivity method in monitoring salt water intrusion. Geophys Geochem Explor. 2021;45(05):1347–53.

[4] Li M, Zhang Z, Yang J, Xie S. Integrated geophysical study in the cemetery of Marquis of Haihun. Archaeol Prospect. 2021;28(4):453–65. doi: 10.1002/arp.1815

[5] Li M, Zhang Z, Liu J. Geophysical regularization of the transcendental petrophysical constraint inversion. Oil Geophys Prospect. 2024;59(6):1420–32.

[6] Athanasiou EN, Tsourlos PI, Papazachos CB, Tsokas GN. Combined weighted inversion of electrical resistivity data arising from different array types. J Appl Geophys. 2007;62(2):124–40. doi: 10.1016/j.jappgeo.2006.09.003

[7] Candansayar ME. Two-dimensional individual and joint inversion of three- and four-electrode array dc resistivity data. J Geophys Eng. 2008;5(3):290–300. doi: 10.1088/1742-2132/5/3/005

[8] Kaufmann O, Quinif Y. An application of cone penetration tests and combined array 2D electrical resistivity tomography to delineate cover-collapse sink holes prone areas. In: Beck BF, Herring JG, editors. Geotechnical and environmental applications of karst geology and hydrology. Lisse: Balkema; 2001. p. 359–64.

[9] Leontarakis K, Apostolopoulos GV. Laboratory study of the cross-hole resistivity tomography: The Model Stacking (MOST) Technique. J Appl Geophys. 2012;80:67–82. doi: 10.1016/j.jappgeo.2012.01.005

[10] Stummer P, Maurer H, Green AG. Experimental design: Electrical resistivity data sets that provide optimum subsurface information. Geophysics. 2004;69:120–39.

[11] Zhou W, Beck B, Adams A. Effective electrode array in mapping karst hazards in electrical resistivity tomography. Environ Geol. 2002;42(8):922–8. doi: 10.1007/s00254-002-0594-z

[12] Bellmunt F, Marcuello A. Method to obtain standard pseudosections from pseudo pole–dipole arrays. J Appl Geophys. 2011;75(3):419–30. doi: 10.1016/j.jappgeo.2011.07.020

[13] Xu B, Noel M. On the completeness of data sets with multielectrode systems for electrical resistivity survey. Geophys Prospect. 1993;41(6):791–801. doi: 10.1111/j.1365-2478.1993.tb00885.x

[14] Lehmann H. Potential representation by independent configurations on a multi-electrode array. Geophys J Int. 1995;120(2):331–8. doi: 10.1111/j.1365-246x.1995.tb01822.x

[15] Blome M, Maurer H, Greenhalgh S. Geoelectric experimental design — Efficient acquisition and exploitation of complete pole-bipole data sets. Geophysics. 2011;76(1):F15–26. doi: 10.1190/1.3511350

[16] Rucker DF. Enhanced resolution for long electrode ERT. Geophys J Int. 2012;191(1):101–11. doi: 10.1111/j.1365-246x.2012.05643.x

[17] Rucker DF, Glaser DR. Standard, random and optimum array conversions from two-pole resistance data. J Environ Eng Geophys. 2015;20(3):207–17. doi: 10.2113/jeeg20.3.207

[18] You Y, Butler SL. Reliable linear transformation of pseudo-pole-pole electrical resistivity datasets. J Appl Geophys. 2024;227:105420. doi: 10.1016/j.jappgeo.2024.105420

[19] Zhang ZY, Li M. Unified framework structure for forward and inversion based on object-oriented ideology. Prog Geophys. 2012;27(3):1207–12.

[20] Butler SL. On the determinability of all of the self‐ and mutual resistances in a grounded electrode array. Geophys Prospect. 2019;67(5):1431–8. doi: 10.1111/1365-2478.12786

[21] Carpenter EW. Some notes concerning the Wenner configuration. Geophys Prospect. 1955;3(4):388–402. doi: 10.1111/j.1365-2478.1955.tb01384.x

[22] Habberjam GM. On the application of the reciprocity theorem in resistivity prospecting. Geophysics. 1967;32(5):918–9. doi: 10.1190/1.1439900

[23] Parasnis DS. Reciprocity theorems in geoelectric and geoelectromagnetic work. Geoexploration. 1988;25(3):177–98. doi: 10.1016/0016-7142(88)90014-2

[24] Van Nostrand RG, Cook KL. Interpretation of resistivity data: USGS. Prof. Pap. 499; 1966.

[25] Cubbage B, Noonan GE, Rucker DF. A modified Wenner array for efficient use of eight-channel resistivity meters. Pure Appl Geophys. 2017;174(7):2705–18. doi: 10.1007/s00024-017-1535-9

[26] Beard LP, Tripp AC. Investigating the resolution of IP arrays using inverse theory. Geophysics. 1995;60(5):1326–41. doi: 10.1190/1.1443869

